# Microglia: A pharmacological target for the treatment of age-related cognitive decline and Alzheimer’s disease

**DOI:** 10.3389/fphar.2023.1125982

**Published:** 2023-02-09

**Authors:** Chloe G. McKee, Madison Hoffos, Haley A. Vecchiarelli, Marie-Ève Tremblay

**Affiliations:** ^1^ Division of Medical Sciences, University of Victoria, Victoria, BC, Canada; ^2^ Department of Biology, University of Victoria, Victoria, BC, Canada; ^3^ Département de Médecine Moléculaire, Université Laval, Québec City, QC, Canada; ^4^ Axe Neurosciences, Centre de Recherche du CHU de Québec, Université Laval, Québec City, QC, Canada; ^5^ Neurology and Neurosurgery Department, McGill University, Montreal, QC, Canada; ^6^ Department of Biochemistry and Molecular Biology, University of British Columbia, Vancouver, BC, Canada; ^7^ Centre for Advanced Materials and Related Technology (CAMTEC), University of Victoria, Victoria, BC, Canada; ^8^ Institute for Aging and Lifelong Health, University of Victoria, Victoria, BC, Canada

**Keywords:** microglia, microglial diversity, cognitive aging, mild cognitive impairment, Alzheimer’s disease, pharmacology

## Abstract

As individuals age, microglia, the resident immune cells of the central nervous system (CNS), become less effective at preserving brain circuits. Increases in microglial inflammatory activity are thought to contribute to age-related declines in cognitive functions and to transitions toward mild cognitive impairment (MCI) and Alzheimer’s disease (AD). As microglia possess receptors for communicating with the CNS environment, pharmacological therapies targeting these pathways hold potential for promoting homeostatic microglial functions within the aging CNS. Preclinical and early phase clinical trials investigating the therapeutic effects of pharmacological agents acting on microglia, including reactive oxygen species, TREM2, fractalkine signaling, the complement cascade, and the NLRP3 inflammasome, are currently underway; however, important questions remain unanswered. Current challenges include target selectivity, as many of the signaling pathways are expressed in other cell types. Furthermore, microglia are a heterogenous cell population with transcriptomic, proteomic, and microscopy studies revealing distinct microglial states, whose activities and abundance shift across the lifespan. For example, homeostatic microglia can transform into pathological states characterized by markers of oxidative stress. Selective pharmacological targeting aimed at limiting transitions to pathological states or promoting homeostatic or protective states, could help to avoid potentially harmful off-target effects on beneficial states or other cell types. In this mini-review we cover current microglial pathways of interest for the prevention and treatment of age-related cognitive decline and CNS disorders of aging focusing on MCI and AD. We also discuss the heterogeneity of microglia described in these conditions and how pharmacological agents could target specific microglial states.

## Introduction

Microglia, the resident immune cell of the central nervous system (CNS), play important roles across health and disease. They defend the CNS against infection and injury (Napoli and Neumann, 2009), contribute to neurogenesis (Sierra et al., 2014), refine synaptic connections (Paolicelli et al., 2011; Tremblay, 2011), and support neurons and other glial cells (Hansson and Rönnbäck, 2003). As microglia perform essential functions, their dysfunction is implicated in nearly all neurological pathologies, including the age-related neurodegenerative condition mild cognitive impairment (MCI), which can transition into Alzheimer’s disease (AD) (Tay et al., 2018). Genome-wide association studies (GWAS) found microglia to most strongly express 60% of single nucleotide polymorphisms (SNPs) associated with AD risk (Zhang et al., 2014; McQuade and Blurton-Jones, 2019). Microglial functions are also affected during normal aging, which describes a process of gradual decline in cognitive functions including reasoning, memory, and processing speed (Harada et al., 2013). Microglia are highly sensitive to their surrounding environment and respond by modulating their morphology and activity (Carvalho-Paulo et al., 2021). Microglia possess various receptors to sense their microenvironment that also regulate their functions (Song and Suk, 2017; Šimončičová et al., 2022), holding potential for the pharmacological treatment and prevention of MCI and AD.

During aging, the CNS environment becomes more inflammatory, triggering microglial reactivity (Norden et al., 2015), dystrophy (Streit et al., 2004), dysfunction (Mosher and Wyss-Coray, 2014), and senescence (Angelova and Brown, 2019). Microglial reactivity alters the balance between pro- and anti-inflammatory cytokine release, which can impair glial and neuronal functions leading to tissue damage. Notable age-related changes in microglia include their increased production of reactive oxygen species (ROS) and deficits in lysosomal digestion (Nakanishi and Wu, 2009). This compromises their maintenance of homeostasis, notably *via* reduced phagocytosis of apoptotic cells, aggregated proteins such as amyloid-beta (Aβ), and myelin debris (Harry, 2013; Rawji et al., 2016; Koellhoffer et al., 2017; Gabandé-Rodríguez et al., 2020). However, microglial phagocytosis is also involved in synaptic loss, a major correlate of cognitive decline hypothesized to be involved in the initiation and acceleration of AD (Terry et al., 1991; Scheff et al., 2006; Herrup, 2015). Thus, pharmacological approaches modulating microglial phagocytosis should consider cargo specificity. Classical pharmacological approaches, such as minocycline, focused on preventing microglial reactivity (Šimončičová et al., 2022) and have limited efficacy likely because they compromise beneficial microglial functions. New therapeutic approaches should promote beneficial microglial functions while preventing or reversing detrimental ones. Considering that microglia are highly heterogeneous, as notably identified by transcriptomic and microscopy studies (Table 1) (Paolicelli et al., 2022), such a goal could be accomplished by targeting specific microglial states which become more abundant with aging and AD pathology.

**TABLE 1:** Microglial states associated with normal cognitive aging and age-related neurodegenerative diseases which are discussed in our mini-review.

Microglial state	Species and model identified in	Method of identification	Genetic markers	Protein markers	Beneficial or detrimental?	Potential pharmacological approaches	Relevant studies
Dark (DM)	Mouse: *Cx3Cr1* KO (3 Mo), APP-PS1 (14 Mo, 20 Mo), C57BL/6J (3 Mo, 14 Mo, 20 Mo) Human post-mortem: aged (81 years), middle-aged (45 years), AD cases	EM	Not yet investigated to our best knowledge	CD11B ↑	**Dependent on context**	Fractalkine supplementation	Bisht et al. (2016)
				IBA1 ↓	Detrimental when over phagocytosing synapses, beneficial when clearing Aβ plaques	Inhibition of the cyclooxygenase pathway to limit ROS production	St-Pierre et al. (2022b)
				4D4 +	↑ iron storage suggesting dysfunctional iron metabolism, possibly contributing to electron-dense appearance	Antibodies targeting complement cascade receptors or proteins	El Hajj et al. (2019)
				TREM2 +			
				P2RY12 –			
				CX3CR1 –			
Lipid-droplet-accumulating (LDAM)	Mouse: *Grn* KO (9-10 Mo), ${Wrn}^{△hel/△hel}$ (14 Mo), C57BL/6J (14 Mo, 20 Mo), CBA/CaJ (20 Mo)	EM	*Cat* ↑	CCL3 ↑	**Detrimental**	Inhibition of the cyclooxygenase pathway to ↓ ROS levels	Marschallinger et al. (2020)
		Transcriptomics	*Kl* ↑	CX3CL10 ↑	Defective in phagocytosis leading to an accumulation of debris, produce ↑ levels of ROS and pro-inflammatory cytokines		Hui et al. (2018)
		Cytokine measurements on isolated microglia	*Ppp1cb* ↑	IL-6 ↑			Tremblay et al. (2012)
			*Rap1b* ↑				
			*Plin3* ↑				
			*Acly* ↑				
			*Clec7a* ↓				
Dystrophic/senescent	Mouse: C57BL/6J (24 Mo)	Transcriptomics	*Apoe* ↑	Ferritin	**Detrimental**	Inhibition of the cyclooxygenase pathway to ↓ ROS production	Streit et al. (2004)
	Human post-mortem: various ages (10–100), AD cases	LM	*B2m* ↑		↓ ability to efficiently migrate to sites of injury, phagocytose debris, and survey the parenchyma		Streit et al. (2009)
			*Cst7* ↑		↑ iron storage, suggesting dysfunctional iron metabolism possibly contributing to ↑ oxidative stress		Shahidehpour et al. (2021)
			*Fth1* ↑				Lopes et al. (2008)
			*Cd11c* ↑				Zhang et al. (2022)
			*Lpl* ↑				
			*Cx3cr1* ↓				
			*P2ry12* ↓				
			*Tmem119* ↓				
White matter associated (WAM)	Mouse: C57BL/6J (18–20 Mo)	Transcriptomics	*Apoe ↑*	Not yet investigated to our best knowledge	**Beneficial**	Agonizing antibodies that ↑ TREM2 signaling	Safaiyan et al. (2021)
		LM	*Trem2 ↑*		Potential to clear degenerated myelin that accumulates in the white matter during aging and neurodegenerative disease	Antibodies that block the shedding of soluble TREM2	
		EM	*Cst7 ↑*				
			*Clec7a ↑*				
			*Bm2 ↑*				
			*Lyz2 ↑*				
			*P2ry12 ↓*				
			*P2ry13 ↓*				
			*Csfr1 ↓*				
			*Cx3cr1↓*				
			*Tmem119 ↓*				
Disease associated (DAM)	Mouse: 5XFAD (1 Mo, 3 Mo, 6 Mo, 8 Mo)	Transcriptomics	*Cx3cr1* ↓	Not yet investigated to our best knowledge	**Beneficial**	Agonizing antibodies that increase TREM2	Keren-Shaul et al. (2017)
	Human post-mortem: AD cases	LM	*P2ry12/13* ↓		Potential to restrict neurodegeneration by phagocytosing debris and Aβ plaques	Antibodies that block the shedding of soluble TREM2	
			*Tmem119* ↓				
			*Apoe* ↑				
			*Trem2* ↑				
			*Cst7* ↑				
			*Lpl* ↑				
			*Clec7a* ↑				

Abbreviations: *CX3CR1* KO, fractalkine signaling deficient mice where the fractalkine receptor, CX3CR1 is mutated; APP-PS1, amyloid-β (Aβ) based mouse model of Alzheimer’s disease (AD) pathology; C57BL/6J, wild-type mice, known to undergo age-related loss of audition; *Grn* KO, mouse model of frontotemporal dementia pathology where exons of the granulin (*Grn*) allele are deleted; ${Wrn}^{△hel/△hel}$ , mouse model of Werner’s syndrome pathology where the helicase domain of the murine WRN orthologue are deleted; CBA/CaJ, wild-type mice, known to undergo age-related loss of vision; 5XFAD, Aβ based mouse model of AD pathology; months (Mo); electron microscopy (EM); light microscopy (LM); reactive oxygen species (ROS); triggering receptor expressed on myeloid cells 2 (TREM2).

**FIGURE 1:**
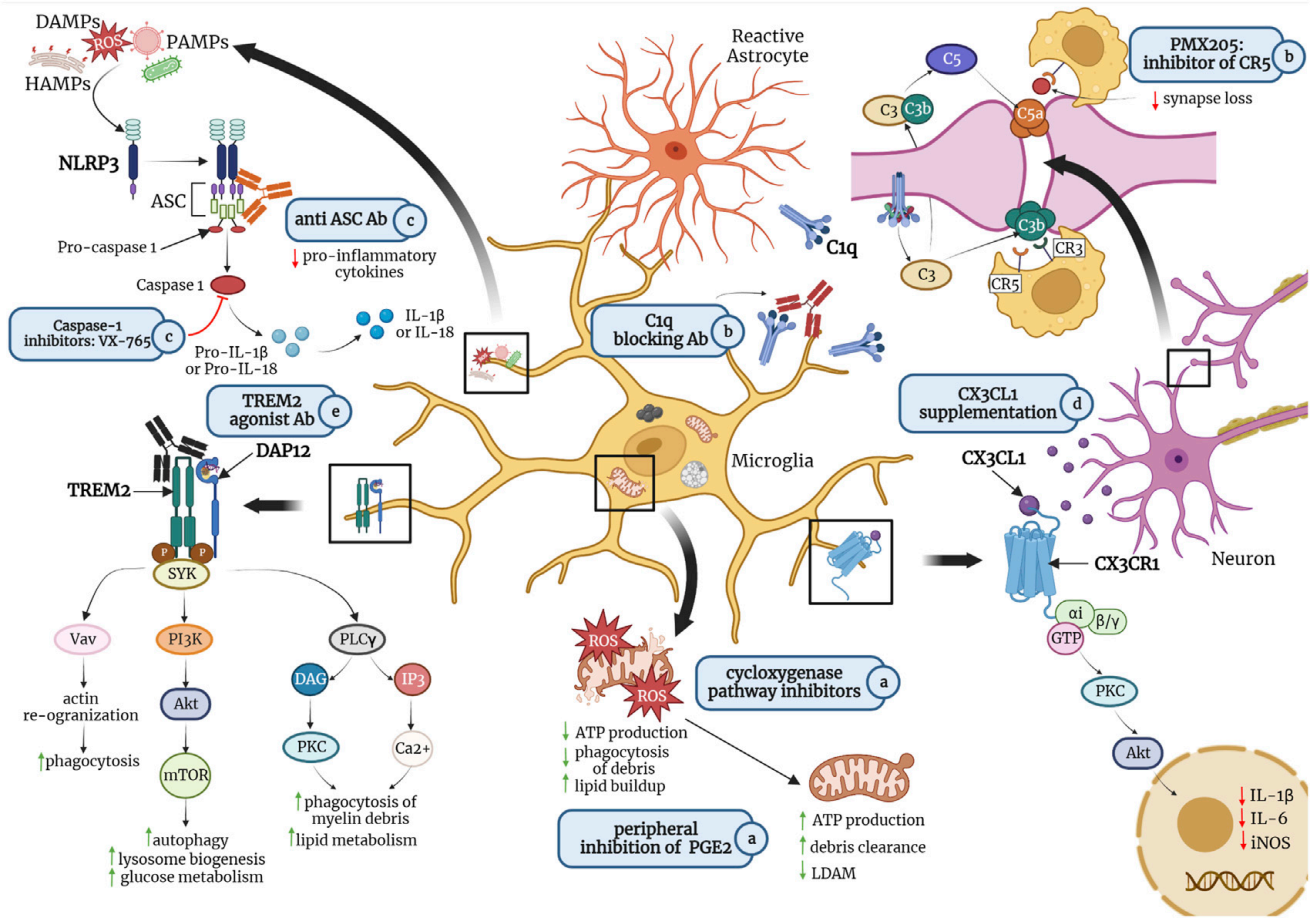
Potential pharmacological treatments for promoting homeostatic microglial states and preventing or reversing detrimental states include a) peripheral inhibition of lipid messenger prostaglandin E2 (PGE2) or cyclooxygenase inhibitors such as non-steroidal anti-inflammatory drugs (NSAIDs) to maintain mitochondrial morphology, thereby increasing adenosine triphosphate (ATP) production and debris clearance; b) antibodies against C1q, the initiating protein in the complement cascade, or small molecule inhibitors of complement receptor 5 (CR5), such as PMX205 to reduce microglia-mediated phagocytosis of synapses; c) caspase-1 inhibitors or antibodies against apoptosis-associated speck-like protein containing a CARD (ASC), a central adaptor protein of the NLR family pyrin domain containing 3 (NLRP3) inflammasome, to inhibit the production of pro-inflammatory cytokines such as interleukin (IL)-1β and IL-18; d) fractalkine (CX3CL1) supplementation to reduce the production of pro-inflammatory cytokines; and e) agonist antibodies targeting triggering receptor expressed on myeloid cells (TREM)2 to promote microglial phagocytosis of myelin debris and amyloid-β (Aβ) plaques, as well as glucose metabolism. Abbreviations: ROS, reactive oxygen species; LDAM, lipid-droplet accumulating microglia; CX3CR1, fractalkine receptor; PKC, protein kinase C; Akt, protein kinase B; iNOS; inducible nitric oxide synthase; IL-6, interleukin 6; HAMPs, homeostasis-altering molecular patterns; DAMPs, danger-associated molecular patters; PAMPs, pathogen-associated molecular patterns; C3, C3 convertase; C3b, complement component 3b (an opsonin); CR3, complement receptor three (binds to C3b); C5, C5 convertase; C5a, complement component C5a (an anaphylatoxin); CR5, complement receptor five (binds to C5a); DAP12, DNAX-activating protein of 12 kDa; SYK, spleen tyrosine kinase; Vav, a guanine nucleotide exchange factor (GEF) for small G proteins of the Rho family; PI3K, phosphatidylinositol-3 kinase; mTOR, mammalian target of rapamycin; PLC**γ,** phospholipase C gamma; DAG, diacylglycerol, IP3; inositol trisphosphate. Figure is not drawn to scale. Created in Biorender.

In this mini-review we cover current microglial pathways which have shown potential for the prevention or treatment of AD and slowing of cognitive aging. We also discuss how pharmacological agents could target specific microglial states to maintain protective functions or prevent detrimental ones during aging (Figure 1). Challenges surrounding microglial pharmacological targeting still remain, such as getting therapeutics across the blood-brain barrier (BBB) (Gabathuler, 2010) and avoiding off-target effects on other CNS cells that express many of the same receptors of interest as microglia.

## Reactive oxygen species

ROS are molecules containing unpaired electrons formed by the partial reduction of oxygen within the mitochondrial electron transport chain (Guo et al., 2013). Under physiological conditions, ROS act as important signaling molecules (Ray et al., 2012); however, their levels increase during aging and in AD (Brieger et al., 2012; Manoharan et al., 2016). When ROS overwhelm antioxidant defense mechanisms, they can damage macromolecules and organelles, such as mitochondria (Guo et al., 2013), where disrupted cristae membranes are a marker of oxidative stress (Picca et al., 2020). Dark microglia (DM), a pathology associated microglial state (Table 1) found in mouse models of aging and AD pathology, and aging human post-mortem samples, often contain mitochondria with swollen and disrupted membranes (Bisht et al., 2016; St-Pierre et al., 2022a).

Lipid-droplet accumulating microglia (LDAM) which increase during aging in mice and humans, produce high levels of ROS and proinflammatory cytokines [e.g., interleukin (IL)-1β, IL-6, and tumor necrosis factor (TNF)α] and have deficits in phagocytosis (Ye et al., 2017). Promoting microglial autophagy, responsible for transporting cytoplasmic organelles to lysosomes for degradation, through *in vitro* treatment with rapamycin, a Food and Drug Administration-approved immunosuppressant with anti-cognitive aging potential (Blagosklonny, 2019), decreased ROS and pro-inflammatory cytokine production (Ye et al., 2017). Thus, increasing microglial autophagy may limit lipid droplet buildup and transitions from homeostatic microglia to LDAM.

Increased signaling through the lipid messenger prostaglandin E2 (PGE2), a downstream component of the cyclooxygenase-2 pathway involved in inflammation was reported in aged mice (Minhas et al., 2021). This pathway converts glucose to glycogen, while reducing glucose flux through glycolysis and the Krebs cycle, thereby decreasing ATP production. Pharmacological inhibition of PGE2 in the periphery restored microglial mitochondrial morphology, ATP production, and long-term potentiation within the hippocampus to young adult levels, while resolving spatial memory deficits (Minhas et al., 2021). Furthermore, long-term treatment with drugs that inhibit cyclooxygenases, key enzymes involved in the production of PGE2, such as non-steroidal anti-inflammatory drugs (NSAIDs), delayed AD onset in pilot clinical trials and decreased microglial reactivity in rodent models of AD pathology (Ferencik et al., 2001; Krause and Müller, 2010).

Studies in models of Aβ pathology including 5XFAD (Baik et al., 2019) and APP/PS1 mice (McIntosh et al., 2019; Guillot-Sestier et al., 2021), have shown that microglia surrounding Aβ plaques shift from oxidative phosphorylation (OXPHOS) to the less efficient glycolytic metabolism. This reduces ATP production, decreases microglial phagocytosis, and leads to increased Aβ accumulation (McIntosh et al., 2019). Additionally, transitions to glycolytic metabolism caused microglia to become more iron retentive (McIntosh et al., 2019), a known trigger of ROS production (Picca et al., 2020). Increased ferritin immunoreactivity was reported in dystrophic and senescent microglia in human post-mortem samples from patients with and without dementia (Lopes et al., 2008). Although more research is needed, iron retention could contribute to the electron-dense appearance of DM. Overall, these findings suggest that reducing oxidative stress and maintaining microglial mitochondrial OXPHOS could help promote transitions from DM and LDAM to homeostatic states, while possibly limiting cognitive decline during aging.

## Complement cascade

The complement cascade is an innate immune system pathway that detects and removes pathogens and dying cells (Ricklin et al., 2010). Within the CNS, neurons, astrocytes, and primarily microglia produce the complement protein C1q (Kouser et al., 2015; Fonseca et al., 2017; Asano et al., 2020). When C1q is deposited onto synapses it acts as an “eat me signal” targeting them for phagocytosis (Hong et al., 2016b) or trogocytosis (Lim and Ruthazer, 2021).

C1q expression is consistently upregulated with age in the mouse and human CNS, especially in the hippocampus (Stephan et al., 2013), and may contribute to the synaptic loss observed during normal aging (Terry et al., 1991; Jackson et al., 2019). Furthermore, Aβ fibrils produce complement proteins in various mouse models of AD pathology (Rogers et al., 1992; Bradt et al., 1998). Knocking out the C3 convertase, located downstream of C1q, is protective against age-dependent synapse and neuron loss within the hippocampus, and results in improved memory and spatial learning in aged wild-type (Shi et al., 2015) and APP/PS1 (Shi et al., 2017) mice. Pharmacological approaches such as small molecule inhibitors and monoclonal antibodies have demonstrated some success in mouse models of AD pathology. Oral administration of PMX205, an antagonist of C5aR, a receptor which triggers microglial phagocytosis, significantly reduced Aβ load in Tg2576 mice (model of Aβ pathology), together with Aβ load and hyperphosphorylated Tau in 3xTg mice (model of Aβ/Tau pathology), compared to untreated controls (Fonseca et al., 2009). In Tau-P301S mice (model of Tau pathology), injection of a C1q-blocking antibody into the hippocampus reduced synaptic markers by 50% within microglial lysosomes, suggesting decreased synaptic phagocytosis (Dejanovic et al., 2018). The broad spectrum humanized monoclonal antibody against C1q, ANX005, is currently undergoing Phase II trials, while its murine precursor ANXM-1 inhibited the complement cascade *in vivo* in wild-type mice centrally receiving Aβ oligomers (Hong et al., 2016a).

In terms of targeting microglial states, DM highly express C3R in their processes surrounding synapses and Aβ (Bisht et al., 2016). Inhibition of the complement cascade could help lessen the pathological synapse loss associated with DM; however, challenges include a lack of specificity as neurons and astrocytes, among other cells, also express complement components (Barnum, 1995).

## NLRP3 inflammasome

The NLRP3 inflammasome is a pattern recognition receptor expressed by many cell types (Holbrook et al., 2021), including microglia (Nayak et al., 2014), that recognizes homeostasis-altering molecular patterns (HAMPs), danger-associated molecular patterns (DAMPs), and pathogen-associated molecular patterns (PAMPs) (Kelley et al., 2019). HAMPs arise from cellular imbalances such as endoplasmic reticulum and Golgi stress (Liston and Masters, 2017), PAMPs are produced by microbial pathogens, and DAMPs by uric acid crystals (Liston and Masters, 2017) and ROS (Kelley et al., 2019), among other signals released during endogenous cell death.

When activated, NLRP3 induces caspase-1-mediated cleavage of IL-18 and IL-1β (Kelley et al., 2019). IL-1β which is elevated in the cerebrospinal fluid (CSF) and brain of patients with AD is considered toxic to microglia (Blevins et al., 2022) by triggering increased oxidative stress and apoptosis (Liu and Quan, 2018). Caspase-1 is also associated with inflammatory and cytoprotective responses that include cell death. Heneka et al. (2013) identified increased caspase-1 expression as an indication that NLRP3 may induce neurodegeneration, in patients with AD and APP/PS1 mice. APP/PS1 mice deficient in NLRP3 or caspase-1 displayed reduced spatial memory loss and IL-1β activation, suggesting beneficial outcomes of targeting caspase-1, or its upstream effectors, in AD.

In terms of targeting, VX-765, a small molecule caspase-1 inhibitor that is BBB permeable and approved for clinical trials, reversed impairment of episodic and spatial memory in J20 *APP* ^Sw/Ind^ mice (model of Aβ pathology) (Sri et al., 2019), while preventing brain inflammation and Aβ build-up (Flores et al., 2018). Another small molecule capsase-1 inhibitor, MCC950, inhibits NLRP3 assembly, and IL-1β and Aβ build-up, while increasing microglial phagocytosis of Aβ (Dempsey et al., 2017). These changes were associated with improved working and recognition memory in APP/PS1 mice (Dempsey et al., 2017). MCC950 also prevented Tau pathology in Tau-P301S mice (Stancu et al., 2019).

Antagonizing antibodies that block the assembly of NLRP3 successfully modified disease outcomes in mouse models of AD pathology. Apoptosis-associated speck-like protein containing a CARD (ASC) is produced by microglia and is a central adaptor protein of NLRP3 that binds to Aβ and aids in the formation of Aβ oligomers (Venegas et al., 2017). Venegas et al. (2017) reported that an anti-ASC antibody prevented Aβ from accumulating in APP/PS1 mice. de Rivero Vaccari et al. (2022) also demonstrated that IC100, an anti-ASC antibody, which is BBB permeable and binds ASC filaments, blocks IL-1β production in human blood cell inflammasome assays. *In vivo* testing of IC100 is warranted to determine its therapeutic potential in treating AD.

In a rat model of AD pathology, systemic injection of mefenamic acid, a NSAID, was found to prevent memory deficits (Daniels et al., 2016). Similar behavioral results and decreased microglial reactivity and IL-1β expression, with IL-1β being a product of the NLRP3 signaling pathway, were observed in 3xTg mice centrally receiving mefenamic acid (Daniels et al., 2016). Thus far, animal models testing NLRP3 pathway inhibitors suggest that such treatments may have pharmacological relevance in reducing cognitive aging and AD pathology within patients.

## Fractalkine signaling

Fractalkine signaling is one of the most important communication pathways between neurons and microglia (Mecca et al., 2018; Angelopoulou et al., 2020). Neurons primarily release the chemokine fractalkine (CX3CL1) which binds to the fractalkine receptor (CX3CR1), expressed primarily by microglia (Pawelec et al., 2020), although this is not universal in the CNS (Hatori et al., 2002). Fractalkine signaling is commonly viewed as an “OFF” switch that maintains microglia in a homeostatic state, by downregulating their production of IL-1β (Bachstetter et al., 2011). CX3CL1 expression decreases across the lifespan, being lower in middle-aged and further in aged *versus* young adult rats (Lyons et al., 2009; Bachstetter et al., 2011). Furthermore, human patients with AD compared to MCI and healthy controls have significantly less CX3CL1 in their CSF (Perea et al., 2018).

Targeting of the fractalkine pathway has demonstrated some success. Aged rats centrally treated with recombinant CX3CL1 exhibited increased hippocampal neurogenesis (Bachstetter et al., 2011), suggesting the potential of such treatments to decrease memory impairment in aging. Treatment with exogenous CX3CL1 attenuated microglial reactivity, by decreasing the expression of major histocompatibility complex class II and CD40 in hippocampal sections from aged rats (Lyons et al., 2009). Targeting the fractalkine pathway to treat AD has been more complex. CX3CR1 deficiency in APPS1 and R1.40 mice, models of Aβ pathology, resulted in reduced Aβ deposition potentially through enhanced microglial Aβ phagocytosis (Lee et al., 2010). mRNA expression of TNFα decreased while IL-1β increased in CX3CR1 deficient APP/PS1 mice. Contrastingly, CX3CR1 deficiency in the hAPP J20 mouse model of Aβ pathology did not significantly affect Aβ deposition and instead upregulated TNFα and IL-6 (Cho et al., 2011). Furthermore, CX3CR1 deficiency exacerbated Tau phosphorylation and worsened learning and memory in these mice, suggesting increased cognitive impairment. Similar results were observed in hTau mice, a model of tauopathy, where CX3CR1 deficiency increased tau phosphorylation and aggregation, and the expression of CD68, a marker of phagocytosis notably expressed by microglia (Bhaskar et al., 2010). Further work on simultaneously reducing Aβ deposition and tau pathology *via* targeting of the fractalkine pathway, combined with other targets, is warranted.

In terms of microglial diversity, CX3CR1 deficient young adult mice display a significantly greater density of DM in the hippocampus compared to age-matched WT controls (Bisht et al., 2016). This suggests that fractalkine supplementation may help to prevent increases in DM known to become abundant during aging and AD pathology. Although this supplementation holds potential as a clinical treatment, for cognitive aging especially, it will be necessary to limit its off-target effects on other cells and prevent potentially deleterious outcomes on tau pathology.

## TREM2

TREM2 is an immunoglobulin superfamily receptor, expressed most prominently in microglia within the CNS (Turnbull et al., 2006). The binding of TREM2 ligands including Aβ, apolipoproteins, phospholipids, and lipopolysaccharides (Kober and Brett, 2017) activates DNAX-activating protein of 12 kDa (DAP12), TREM2’s binding partner, increasing microglial migration, lipid metabolism, autophagy, and phagocytosis, notably of myelin (Mazaheri et al., 2017; McQuade et al., 2020; Qu and Li, 2021) and synapses (Filipello et al., 2018). GWAS revealed TREM2 SNPs to increase AD risk by 2–4 times (McQuade and Blurton-Jones, 2019), in addition to accelerating cognitive decline (Rajagopalan et al., 2013; Replogle et al., 2015). Knocking out TREM2 in PS2APP mice, a model of Aβ pathology, impairs microglia-mediated compaction of Aβ into dense plaques (Meilandt et al., 2020).

Activation of the program that leads to disease-associated microglia (DAM) and white-matter associated microglia (WAM), neuroprotective states found in AD and aging, respectively, requires TREM2 signaling. Pharmacological approaches aimed at increasing TREM2 signaling and promoting transitions to DAM hold potential for treating AD. Wang et al. (2020) found that the anti-human TREM2 agonistic antibody, AL002c, decreased filamentous plaques and neurite dystrophy in 5XFAD mice carrying the R47H TREM2 variant associated with AD risk. The clinical variant of AL002c, AL002, was found to be safe and well tolerated in a phase I clinical trial (NCT03635047) (Wang et al., 2020). Other antibody approaches include the monoclonal antibody, 4D9, which promotes TREM2 signaling by blocking shedding of soluble TREM2 (Schlepckow et al., 2020). 4D9 increased microglial phagocytosis of myelin debris and Aβ *in vitro*, while reducing amyloidogenesis in the APP-NL-G-F knock-in mouse model of Aβ pathology (Schlepckow et al., 2020). Increasing TREM2 signaling has the potential to limit the accumulation of lipid droplets and myelin debris which are thought to contribute to LDAM emergence (Marschallinger et al., 2020).

Although modulation of the TREM2 pathway could promote homeostatic microglial states, consideration of disease stage may be important. Work in Psen1 mice, a model of Aβ pathology, found that knockdown of TREM2 in the early to middle stages of AD was beneficial as it inhibited microglial phagocytosis of synapses, while this same approach in middle to late stages was detrimental by impairing microglial phagocytosis of Aβ (Sheng et al., 2019). Future work targeting TREM2 will need to balance promoting microglial phagocytosis of myelin debris and Aβ, while limiting synapse loss, perhaps in combination with other microglial targets.

## Conclusion

Microglia are a heterogenous population whose morphology and activity shifts across the lifespan and between health and disease contexts (Grabert et al., 2016; Hammond et al., 2019). Although more research is needed to elucidate the functions of different microglial states, the diversity of this cell population holds selective pharmacotherapeutic potential for the prevention and treatment of cognitive aging, MCI, and AD. It will be important to identify differences in expressed receptors and signaling pathways, but also specific activities such as phagocytosis, metabolism, and inflammatory mediator production between microglial states (Šimončičová et al., 2022). This could allow for the design of drugs or antibody-mediated therapies that target specific microglial states, while preserving others and their critical functions.

However, several challenges remain. None of the receptors and associated pathways discussed herein or elsewhere (Rock and Peterson, 2006; Song and Suk, 2017; Šimončičová et al., 2022) are microglia-specific. Outstanding obstacles include getting therapeutics across the BBB and translation of the findings from animal models of AD pathology to human clinical trials (Shineman et al., 2011; Banik et al., 2015). Translation issues may be explained by genetic and epigenetic variations across patient profiles (Neuner et al., 2019), differences between human and murine microglia (Abels et al., 2021; Yvanka de Soysa et al., 2022), and the current inability of animal models to capture the complex pathology underlying AD including environmental risk factors (Bales, 2012; Onos et al., 2016). As the field evolves to tackle such issues, exploring microglial diversity will be an essential step to design more selective and effective therapeutics for treating and preventing AD and for slowing the overall cognitive aging process.
